# A comparative evaluation of quality and depth of learning by trainee doctors in regional, rural, and remote locations

**DOI:** 10.1186/s12909-023-04175-7

**Published:** 2023-04-05

**Authors:** Louise Young, Emily Anderson, Tiana Gurney, Lawrie McArthur, Matthew McGrail, Belinda O’Sullivan, Aaron Hollins

**Affiliations:** 1 College of Medicine and Dentistry, James Cook University, Queensland, Australia; 2 Rural Clinical School, The University of Queensland, Queensland, Australia; 3 General Practice Training, James Cook University, Queensland, Australia

**Keywords:** Australia, General Practice, Medical Education

## Abstract

**Background:**

An equitable supply and distribution of medical practitioners for all the population is an important issue, especially in Australia where 28% of the population live in rural and remote areas. Research identified that training in rural/remote locations is a predictor for the uptake of rural practice, but training must provide comparable learning and clinical experiences, irrespective of location. Evidence shows GPs in rural and remote areas are more likely to be engaged in complex care. However, the quality of GP registrar education has not been systematically evaluated. This timely study evaluates GP registrar learning and clinical training experiences in regional, rural, and remote locations in Australia using assessment items and independent evaluation.

**Methods:**

The research team retrospectively analysed GP trainee formative clinical assessment reports compiled by experienced medical educators during real-time patient consultations. Written reports were assessed using Bloom’s taxonomy classified into low and high cognitive level thinking. Regional, rural, and remotely located trainees were compared using Pearson chi-squared test and Fisher’s exact test (for 2 × 2 comparisons) to calculate associations between categorical proportions of learning setting and ‘complexity’.

**Results:**

1650 reports (57% regional, 15% rural and 29% remote) were analysed, revealing a statistically significant association between learner setting and complexity of clinical reasoning. Remote trainees were required to use a high level of clinical reasoning in managing a higher proportion of their patient visits. Remotely trained GPs managed significantly more cases with high clinical complexity and saw a higher proportion of chronic and complex cases and fewer simple cases.

**Conclusions:**

This retrospective study showed GP trainees in all locations experienced comparable learning experiences and depth of training. However, learning in rural and remote locations had equal or more opportunities for seeing higher complexity patients and the necessity to apply greater levels of clinical reasoning to manage each case. This evidence supports learning in rural and remote locations is of a similar standard of learning as for regional trainees and in several areas required a superior level of thinking. Training needs to seriously consider utilising rural and remote clinical placements as exceptional locations for developing and honing medical expertise.

**Supplementary Information:**

The online version contains supplementary material available at 10.1186/s12909-023-04175-7.

## Background

Ensuring an equitably distributed and sustainable rural workforce remain critical challenges for Australia [1]. The aims of its recent National Medical Workforce Strategy 2021–2031 are to rebalance the supply and distribution of medical practitioners, especially generalist doctors (working at a wider scope of practice), to serve the needs of smaller rural and remote communities (Department of Health, 2021). Training in rural and remote locations needs to be increased for this to occur as other research shows duration and continuity of rural learning opportunities during training predict uptake of rural work [2–4] and rural skills development [5]. However, a counter issue is ensuring that medical training is of high quality in all locations.

There is a misconception of rural places being perceived as inferior and static, with the discourse favouring urban places as privileged and superior[6]. Moreover, it is believed that superior medical care, which is structured around urban ideologies of having increments of specialist learning experiences accessible to learners, is a better model and therefore improves the doctor’s learning and development of capabilities [7]. This discourse assumes rural areas offer less specialist (higher order) opportunities and thereby may produce less learning and capability development. It follows that some will be discouraged from pursuing training in the rural/remote areas in the belief that their training in these locations may be inferior to more urban locations [8].

Previous research has shown the benefits of rural experiences for undergraduate medical students with rural placements providing high quality training and educational achievement. In addition exposure to longitudinal clerkships predominantly in smaller community settings over hospital block rotations as students may lead to graduating doctors who are more willing to work remotely once qualified [4, 9–11].

However, to justify learning specifically in rural and remote areas at the postgraduate stage of medical training, there is a need for comparative research about the progress and outcomes of learning in different settings. There have not been any rigorous and systematic evaluations of the value of General Practice (GP) learning for registrars in more remote locations to date, though one study about GP learning experience in smaller rural towns, from the perspective of the GP supervisors, found these contexts do provide rich learning [8].

### Measuring quality of learning: Bloom’s taxonomy

Bloom’s taxonomy is an educational framework for describing increasingly complex cognitive thinking first described by Bloom and his colleagues and later updated by Krathwohl et al. (2001). It involves six levels of thinking classification from simple to complex and concrete to abstract. The cognitive levels are Knowledge, Comprehension, Application, Analysis, Synthesis and Evaluation. This framework or taxonomy was originally developed to be used in the education of school age children; however it is now applied at all levels of education including tertiary education and Continuing Professional Development [12]. As trainee doctors are learning through vocational-based training rather than in educational institutions or classrooms, Bloom’s taxonomy is suitable for application to learning that occurs during clinical training. Bloom’s taxonomy covers dimensions of both knowledge, skills and clinical decision making (and its higher cognitive levels have been found to be representative of clinical reasoning [13, 14].

This study applied Bloom’s taxonomy as the educational framework to explore the depth and quality of GP learning in different geographical locations [15]. The six levels of Bloom’s taxonomy comprise descriptors of cognitive processes used by trainees as learners in working with knowledge and information obtained in each clinical encounter with a patient [GP-16]. The application of Bloom’s taxonomy was used to investigate the thinking skills and diagnostic reasoning required in each patient consultation, diagnosis, treatment, and management plan by GP trainees as outlined in Appendix 1. The current study aims to explore and compare the depth and quality of GP trainee learning in regional, rural, and remote locations.

## Methods

### Setting

**Fig. 1:**
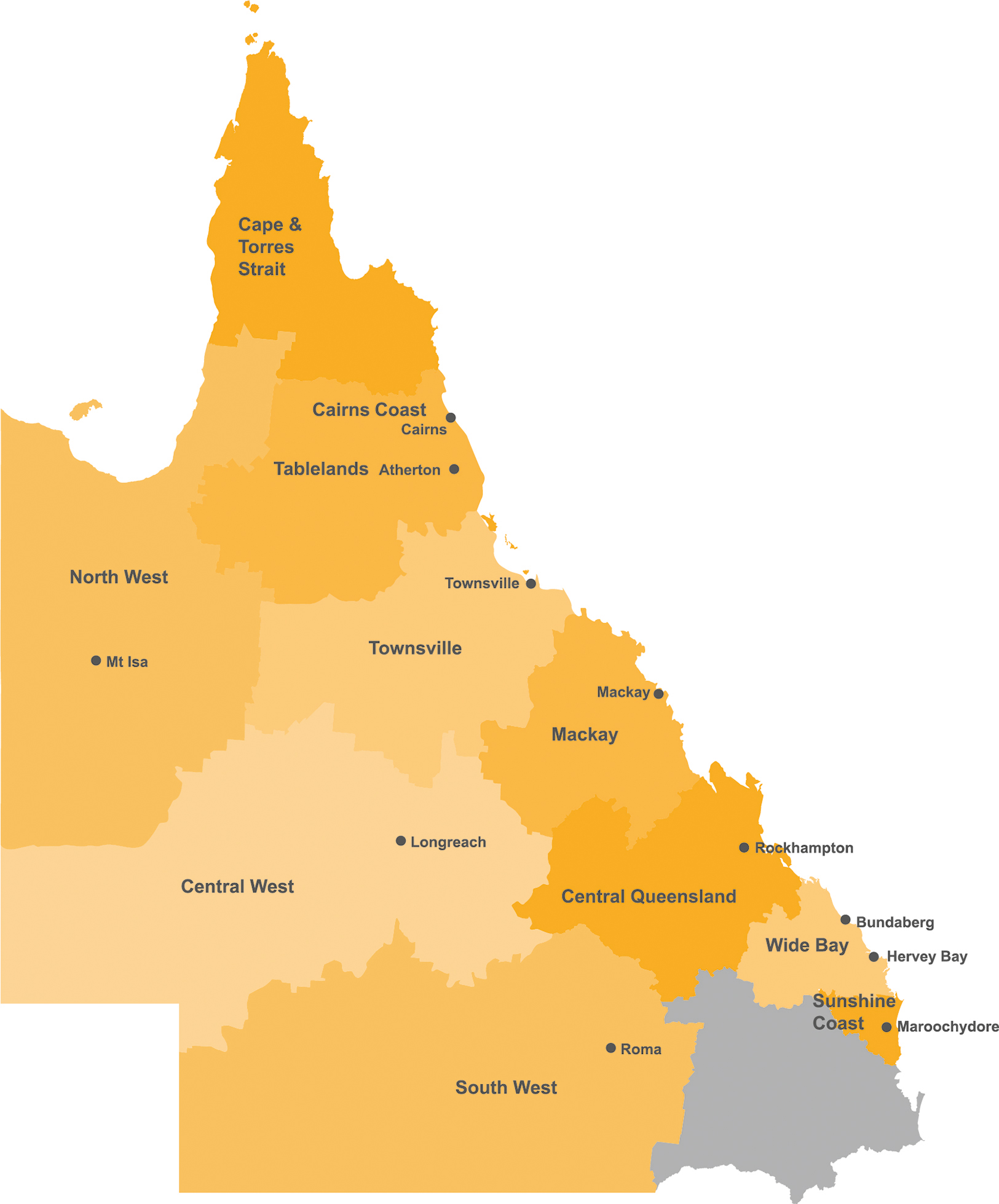
*Coverage of GP training locations across Queensland*

This study was based in the state of Queensland, Australia, covering around 90% of its area, notably excluding the heavily populated south-east corner that includes its capital city of Brisbane (Fig. 1). Queensland has an area of 1,727, 000 square kilometers and is nearly five times the size of Japan and seven times the size of Great Britain. Half the population (approx. 2.5 million) lives outside of Brisbane in regional, rural and remote locations, with first nations people making up over 8% of Queensland’s non-metropolitan population and over 28% of its remote population [17]. Included locations were all remaining communities supporting in-place GP training, with GPs in Australia completing at least two years of their training in community settings including general practices and small rural hospitals.

### Classifying leaning context

Different locations of learning (settings) were allocated as one of three categories for this study, defined in two steps. Step one applied the Modified Monash Model [18] (MMM) rurality classification, with MMM1-2 (metropolitan and regional centres) defining ‘regional’, MMM3-4 (large and medium sized rural towns) defining ‘rural’, and MMM5-7 (small rural towns and remote/very remote communities) defining ‘remote’. Step two involved two trained medical educators, who were familiar with all training locations, assessing the learning context of each community to explore whether this setting involved functioning at scope of rural practice. This was informed by the scope of rural generalist GPs which has been defined in the literature [19, 20]. From this, five locations that were initially classified ‘remote’ were reclassified as ‘rural’, including because it was part of an outreach learning experience, rather than a residential experience in that setting. Additionally, 56 ‘rural’ locations were reclassified as ‘remote’ if trainees resided in-situ full time, worked at generalist scope, did not regularly meet their supervisor face-to-face and thus relied on distant education. This two-step method ensured that the rural location of learning settings was informed by national standards for rurality as well as functional aspects of the context of practices and how doctors worked in those practices.

### Procedure

The application of clinical reasoning by the GP trainee in a patient consultation involves all six levels of Bloom’s taxonomy: (1) knowledge: recall of specific facts related to a case; (2) comprehension: knowing which knowledge is relevant to a case; (3) application: applying patient information to clinical knowledge; (4) analysis: using information and deciding which components require additional information; (5) synthesis: which pieces of information are relevant to the case and which are not relevant; and (6) evaluation: making a judgement or decision about a diagnosis and treatment/management plan.

As higher cognitive level thinking is associated with clinical reasoning [13, 21] it is to be expected that scores reflecting higher cognitive level thinking by GP trainees is aligned with a training experience that is more conducive to learning skills related to clinical reasoning. It is accepted however that this may not take into account pattern recognition processes that the trainees will begin to use in their day-to -day work. In this study the Bloom’s taxonomy levels are used as an educational framework to determine if low or high levels of cognitive processing are being applied by GP trainees in a session of observed patient consultations.

The External Clinical Teaching (ECT) visit is an opportunity for trainees to be observed by an external medical educator assessor whilst trainees consult with real patients. Australian GP trainees receive up to five ECT visits during two years of training towards fellowship of either GP training organisation RACGP or ACRRM. Three visits occur during the first year of community practice when the GP trainees first move to community-based general practice or work in small rural hospitals, two in the first six months and one in the second part of the year. Each ECT visit consists of the assessor observing the trainee for 6–10 random consultations during one session, as well as meeting separately with each trainee’s supervisor. The assessor completes a feedback sheet for each consultation, as an aggregate assessment across specific learning objectives as well as overall feedback relating to progress for each observed trainee. Data from all ECT visit reports that occurred in the first year of community practice in 2019 were included in this study. The study utilised a multi-pronged approach by analysing three components of the ECT visit data for ratings of case complexity based on Bloom’s taxonomy, clinical reasoning ratings (low, medium, high complexity) for the type of case seen as annotated by the medical educator and the level of clinical complexity (acute, chronic, complex, simple) for each case. From the ECT visit consultation reports, descriptions of each observed case written by the medical education assessors, were coded by the researchers (LY, TG, BoS, EA) by main presentation complexity as acute, chronic, complex, or simple across regional, rural, and remote locations of training (Table 1).

**Table 1:** Descriptors for complexity of patient presentation

Presentation Complexity	Descriptors
Acute	new and potentially infectious, inflammatory, and life-threatening until investigated and diagnosed, can be on back of a chronic issue
Chronic	anything the patient and/or doctor knew about already, that is for ongoing management, or risk factors and longstanding conditions (mental, physical) or ageing
Complex	multiple issues of medical and psychosocial nature beyond simply chronic disease management or managing one risk factor
Simple	episodic care / follow-up, prescriptions, or medical/Centrelink forms - something that only needs one appointment to do

Data from each consultation were then classified as having involved either low or high levels of cognitive thinking by applying Bloom’s taxonomy (Appendix 1), to case descriptions written in the ECT visit reports. Low level cognitive thinking was defined as written ECT visit report descriptions with ratings from the first three Bloom cognitive descriptors i.e. Knowledge, Comprehension, and Application, while high level cognitive thinking was scored when the Bloom’s levels of Analysis, Synthesis and Evaluation were rated. Classification was completed independently by three researchers (LY, EA, TG) after training by LY, an experienced medical educator and qualitative researcher. Researchers referred to lists of verbs for each of the Bloom’s levels when deciding on each cognitive rating (see Appendix 2) as well as considering the overall case complexity. Only two cognitive levels (low, high) were used for this rating so there was a clear distinction between the two levels of clinical reasoning and so researchers avoided scoring in the middle. Researchers were randomly allocated trainee ECT visit reports grouped by regional, rural, and remote setting of work but were blinded to the setting when classifying a Bloom’s rating for each trainee. 25% of all data were double coded for reliability with a concurrence of 95% between classifications confirmed at team data meetings.

Written notes by the medical educator assessor in the ECT visit report were examined and recorded according to whether the assessor documented each case as involving low, medium, or high clinical complexity (Table 2). As a measure of validity, the researchers were blinded to these medical educator assessor ratings of case complexity when completing their case complexity ratings using the Bloom’s framework. These ratings were also discussed in the data meetings by the researchers when examining the double-coding outcomes for Bloom’s Taxonomy and were found to be congruent.

**Table 2:** Clinical complexity from ECT visit report and Bloom’s framework

ECTV Assessor ratings of Clinical Complexity	Definition
**Low**	Straightforward clinical problem e.g., repeat script, referral letter, or medical certificate
**Medium**	One or more problems managed in a consultation that requires history, examination and subsequent clinical reasoning
**High**	Complex case requiring high level of clinical reasoning or therapeutic reasoning given comorbidities
**Researcher ratings of Clinical Complexity**	**Applicable Bloom’s level**
**Low**	Knowledge, Comprehension, Application
**High**	Analysis, Synthesis, Evaluation

Ethical approval was obtained from James Cook University Human Research Ethics Committee (H8241) and ratified by The University of Queensland Human Research Ethics Committee (No.2,020,002,764). The need for written informed consent was waived by James Cook University Research Ethics Committees and then ratified by The University of Queensland Human Research Ethics Committee due to the retrospective nature of the data. In addition, trainees were emailed and given the opportunity to remove their data from this study (two trainees opted out). All methods were performed in accordance with the relevant guidelines and regulations by including a statement in the Ethics approval.

James Cook University Institution’s Privacy Officer approved access as per institution policy prior to ethical approval being sought.

Pearson chi-squared test and Fisher’s exact test (for 2 × 2 comparisons) were used to calculate associations between categorical proportions of learning setting and ‘complexity’. All analyses used Stata SE 15.1 for Windows (Stata Corp, College Station, TX, USA) and statistical significance was p < 0.05.

## Results

In this study, 1650 ECT visit reports were analysed, with 57% observed in regional locations, 15% in rural and 29% in remote. There was a statistically significant association between learner setting and complexity of clinical reasoning using Bloom’s taxonomy (p = 0.043). Table 3 reveals that GP trainees in the remote group were most likely required to use a high level of clinical reasoning in managing a significantly higher proportion of their ECT visit patients (17.6%), compared with either those in rural (11.7%) or regional (13.3%) settings.

**Table 3:** GP trainee performance ratings by Bloom’s taxonomy, case complexity and case type by location

	Regional	Rural	Remote	Overall Significance
Bloom’s taxonomy complexity rating				
Low level rating	802 (86.7%)	211 (88.3%)	388 (82.4%)	
High level rating	123 (13.3%)	28 (11.7%)	83 (17.6%)	p = 0.043
Total (Note: n = 15 with Bloom’s value missing)	925	239	471	
p-value	0.031	0.041	N/A	
**Medical Educator assessor ECTV rating by case complexity**				
Low complexity	390 (42.5%)	83 (35.8%)	145 (31.5%)	
Medium complexity	451 (49.1%)	121 (52.2%)	241 (52.3%)	
High complexity	77 (8.4%)	28 (12.1)	75 (16.3%)	p < 0.001
Total (Note: n = 39 with ME complexity value missing)	918	232	461	
p-value	< 0.001	0.258	N/A	
**ECTV patient case type**				
Acute	336 (35.9%)	94 (39.2%)	154 (32.6%)	
Chronic	152 (16.2%)	44 (18.3%)	124 (26.2%)	
Complex	129 (13.8%)	14 (5.8%)	58 (12.3%)	p < 0.001
Simple	320 (34.2%)	88 (36.7%)	137 (29.0%)	
Total	937	240	473	
p-value	< 0.001	= 0.001	N/A	

For all settings approximately half of the ECT visit presentations were rated as medium complexity (Table 3). However, the remote group had significantly more high case complexity ratings (16.3%) when compared to high complexity ratings for regional (8.4%) and for rural (12.1%) trainees (Pearson chi-squared test, p < 0.001). Low case complexity was least common in remote areas (31.5%, compared with 35.8% for rural and 42.5% for regional trainees).

Remote GP trainees saw a higher proportion of chronic presentations (26.2%) compared to trainees in regional (16.2%) and rural (18.3%) settings. Remote trainees also saw a lower proportion of patients with simple and acute presentations (Table 3). In combination, remote GP trainees saw a significantly higher proportion (38.5%) of cases rated as either complex or chronic, compared with 24.1% of cases for rural trainees (p = 0.001) and 30.0% for regional trainees (p < 0.001).

## Discussion

Overall, results from this retrospective study show GP trainees learning in rural and remote locations had equal or more opportunities for higher complexity patients and the necessity to apply greater levels of clinical reasoning to manage each case. This evidence supports that learning in rural and remote locations is of a similar standard of learning as for their regional counterparts. Remote GP trainees were learning from a significantly higher proportion of patient cases rated by medical educators as medium or high complexity which involved having to apply greater levels of clinical reasoning. These trainees had a broader scope of practice seeing more chronic and complex patients and in addition they were rated by medical educators as seeing more complex patients than their rural and regional counterparts. These findings mirror a previous study undertaken in 2003 showing that for non-metropolitan GPs the more rural or remote the practice location the more likely they are to be involved in providing complex care.

Bloom’s taxonomy further supports parity of learning showing that trainees were required to apply higher levels of cognitive thinking and clinical reasoning in patient consultations during observed ECT visits. Remote locations fostered higher cognitive level thinking as outlined by Bloom’s levels, which in turn is evidence of a higher level of clinical reasoning by trainees in these locations. Hence the type of patient consultations that trainees were required to undertake assisted them in learning clinical reasoning skills. Previous studies [22–26] have shown an increase in depth and breadth of clinical skills reported by doctors undertaking education in rural locations.

This study’s positive results support future research being undertaken to better understand the value and benefits of learning in a range and variety of GP training placements. When training involves appropriate experiences, variety, and complexity in patient presentations, as well as good supervision it should not be constrained by location or traditional training approaches. However more detailed studies are required into specific characteristics of locations and learning opportunities which benefit GP trainee learning and the development of clinical reasoning. This study was undertaken with retrospective data using existing assessment and evaluation instruments not designed by the research team and future research might develop projects to confirm these hypotheses. Evaluating additional strategies to overcome the current maldistribution of the medical workforce is also required.

The National Medical Workforce Strategy [27] has a key priority to address the geographic distribution of Australia’s medical workforce and develop more localised training pathways instead of focussing postgraduate training in the capital cities. Results from our project indicate that remote and rural clinical placements are equivalent to and in many situations more beneficial for the learning of clinical reasoning for GP trainees who see a greater range of patients with complex and chronic medical conditions in rural and remote locations. Given that Australia’s population is aging and chronic illnesses are increasing [28], it appears that GPs trained in rural and remote locations are well suited to learn from and manage the health needs of this population into the future. This study provides evidence that rural and remote training locations are preparing GP trainees for this work. Given the maldistribution of the medical workforce in Australia, regular placement of GP trainees in rural and remote locations may assist with the provision of a regular medical workforce for underserved locations as well as providing comparable training.

There are several limitations to this study. Patient allocations during ECT visits were random and so there was no opportunity to have all patient consultations being equivalent cases. As the ECT visits were pre-arranged, registrars were prepared for them at that time, and there is an expectation that the patient mix for each session was random, thus representative of whoever walks through the door of a typical workday. There is no way of confirming that this always occurred.

As this was a retrospective study all data were analysed post ECT visit, and medical educators were not aware their reports were to be used in this way. Some reports were brief with missing information (for assessing clinical reasoning) whilst others wrote a comprehensive report. We only rated the complexity and range of cases based on explicit written information in the reports and did not make any inferences about missing information. However, the large number of ECT visit reports assessed and rated across the range of locations, coders who were blinded to location and double rating of 25% of all cases suggests a high level of rigour and robustness in the research design and thus confidence in the results obtained.

## Conclusion

GP trainee placements in rural and remote locations are beneficial for learning clinical skills and clinical reasoning by offering a diverse range of patient complexities and medical conditions. Our results show that rural and remote training in MMM 3–7 locations is equivalent, if not superior, to some training in MMM1 and 2 locations. There is also the added benefit for populations in these rural and remote underserved locations in having access to regular and reliable medical expertise which may help alleviate some of the maldistribution of the Australian medical workforce. Training for specialty medical practice needs to seriously consider rural and remote clinical placements as exceptional locations for developing and honing medical expertise.

## Electronic supplementary material

Below is the link to the electronic supplementary material.

**Appendices**: Appendix 1 Bloom’s taxonomy levels of thinking vs GP knowledge/skill comparison. Appendix 2: Bloom’s taxonomy and cognitive rating

## Data Availability

The data that support the findings of this study are available from JCUGP Training, but restrictions apply to the availability of these data, which were used under license for the current study, and so are not publicly available. Data are however available from the authors upon reasonable request.
