# Supplementary material for: A comparative evaluation of quality and depth of learning by trainee doctors in regional, rural, and remote locations
Source: BMC Med Educ. 2023 Apr 5;23:215. doi: 10.1186/s12909-023-04175-7 (PMC10077336; doi:10.1186/s12909-023-04175-7)
Supplement: Supplementary file 1 — Appendices: Appendix 1 Bloom’s taxonomy levels of thinking vs GP knowledge/skill comparison. Appendix 2: Bloom’s taxonomy and cognitive rating [file 12909_2023_4175_MOESM1_ESM.docx]

**Appendices**

**Appendix 1 Bloom’s taxonomy levels of thinking vs GP knowledge/skill comparison**

| **Bloom’s level** | **Characteristics** | **Skill** | **Clinical example** |
| --- | --- | --- | --- |
| Knowledge | Recall facts and basic concepts | What is the anatomy of? What medication is used for? | Causes of lumps in breast |
| Comprehension | Use or interpret information/knowledge in new situations | What is a safe use of blood pressure medication for this patient?  What is the important information for this patient? | Describe why this patient should be referred for a mammogram based on their presenting information |
| Application | Use diagnostic and examination information to generate differential diagnoses | Ordering and interpreting diagnostic tests to differentiate the problem. How would results show if patient was older/younger, different gender, Indigenous? | Using the palpation, ultrasound, and the mammogram to decide whether a person has a cyst, fibroadenoma or a cancerous lump |
| Analysis | Draw connections between clinical knowledge and patient history and individual characteristics. | What is the diagnosis/treatment/ management/likely outcome for this patient? What information is missing? | Explore whether this patient should also have a biopsy based on the diagnostic information and what additional information the patient is reporting. |
| Synthesis | Draw connections between diagnostic information and the patient history. Refine diagnoses | What do these findings mean?  What are the relevant pieces of clinical information? | Interpreting the biopsy, considering the patient’s family history, and refining diagnoses |
| Evaluation | Generate a diagnosis and decide on treatment/management plan? | How well have you managed the patient?  New patient, minimal supervision | Decide on how to refer and manage the patient’s breast cancer |

**Appendix 2: Bloom’s taxonomy and cognitive rating**

| **Bloom’s level** | **Characteristics** | **Verb Examples** | **Cognitive Thinking Rating Allocated by Researchers** |
| --- | --- | --- | --- |
| **Knowledge** | Rote recall /re-state facts without understanding. Exhibits previously learned material by recalling facts, terms, basic concepts and answers | Choose  Recognise  Repeat  Show  Tell  Recite | Low |
| **Comprehension** | To show understanding finding information from the text. Demonstrating basic understanding of facts and ideas. | Ask  Compare  Summarise  Discuss  Demonstrate  Predict | Low |
| **Application** | To use in a new situation. Solving problems by applying acquired knowledge, facts, techniques and rules in a different way. | Act  Link  Interpret  Teach  Practice  Model | Low |
| **Analysis** | To examine in detail. Examining and breaking information into parts by identifying motives or causes; making inferences and finding evidence to support generalisations. | Investigate  Question  In-depth discussion  Test for  Appraise | High |
| **Synthesis** | To change or create into something new. Compiling information together in a different way by combining elements in a new pattern or proposing alternative solutions. | Compile  Plan  Revise  Simplify  Think  Integrate | High |
| **Evaluation** | To justify. Presenting and defending opinions by making judgements about information, validity of ideas or quality of work based on a set of criteria. | Prioritise  Select  Validate  Choose  Determine | High |
